# Inflammation- and regulated-cell-death–relevant transcriptomic signatures in bladder urothelial carcinoma: cross-cohort reproducibility, exploratory single-cell localization, and *in vitro* qRT-PCR validation

**DOI:** 10.3389/fcell.2026.1986148

**Published:** 2026-10-28

**Authors:** Wenbin Wang, Xian Zhang, Zhijia Li, Daya Wang, Huiyang Cai

**Affiliations:** 1 Department of Urology, Wenzhou Central Hospital, Affiliated to Wenzhou Medical University, Wenzhou, Zhejiang, China; 2 Department of Anesthesiology, The First Affiliated Hospital of Wenzhou Medical University, Nanbaixiang, Wenzhou, Zhejiang, China

**Keywords:** bladder urothelial carcinoma, epithelial–mesenchymal transition, inflammation, qRT-PCR, regulated cell death, single-cell RNA sequencing, transcriptomic signature, tumor microenvironment

## Abstract

**Background:**

Bladder urothelial carcinoma develops within an immune-active, metabolically stressed, and tissue-remodeling microenvironment in which regulated cell-death pathways may interact with inflammatory signaling. We evaluated whether tumor-associated transcriptional alterations are reproducible across independent cohorts and whether selected candidates can be localized at single-cell resolution and supported by *in vitro* expression validation.

**Methods:**

TCGA-BLCA (412 primary tumors and 19 solid-tissue normal samples) and GSE13507 (165 primary tumors and 9 normal bladder mucosa controls) were analyzed independently for differential expression. A 15-gene tumor-upregulated transcriptional signature derived in TCGA was evaluated in GSE13507 without gene re-selection. Five pure-urothelial-carcinoma tumor regions from three patients in GSE293189 were analyzed exploratorily by single-cell RNA sequencing. ULBP1, IL37, COL10A1, COL11A1, and IGF2BP3 were further assessed by qRT-PCR in SV-HUC-1, T24, 5637, and UM-UC-3 cell lines.

**Results:**

Across 16,759 matched genes, cohort-specific effects were positively correlated (Spearman ρ = 0.58, P < 1 × 10^−300^), and 4,102 genes were significant in both cohorts with concordant direction. The TCGA-derived signature was higher in tumors than normal tissues and varied across reported pathologic stages, although the highly unbalanced stage distribution precluded interpretation as a clinical stage classifier. Single-cell analysis retained 1,512 cells and resolved epithelial/tumor-associated, myeloid, T-cell, B-cell, NK-cell, and fibroblast compartments with distinct basal, luminal, EMT, and immune states. All five qRT-PCR candidates were consistently upregulated in bladder-cancer cell lines relative to SV-HUC-1.

**Conclusion:**

The study identifies a cross-cohort reproducible tumor-associated transcriptional signal and places selected candidates within a heterogeneous cellular context. qRT-PCR provides orthogonal expression-level support for selected candidates, but neither transcriptomic nor qRT-PCR data alone establish functional execution of apoptosis, necroptosis, pyroptosis, or ferroptosis.

## Introduction

Bladder cancer is a clinically heterogeneous malignancy spanning recurrent non-muscle-invasive disease and biologically aggressive muscle-invasive or metastatic states. Its management increasingly integrates molecular information with surgery, intravesical therapy, cytotoxic chemotherapy, immune checkpoint blockade, antibody–drug conjugates, and targeted agents, yet reliable biomarkers for disease stratification remain limited (Lenis et al., 2020; Kamat et al., 2016; Powles et al., 2022; Robertson et al., 2017). Urothelial carcinoma is also highly immunogenic. Tumor cells, infiltrating leukocytes, stromal populations, extracellular-matrix components, and soluble mediators interact to generate a microenvironment in which inflammation can simultaneously support antitumor immunity and promote progression, immune escape, and epithelial plasticity (Greten and Grivennikov, 2019; Martins-Lima et al., 2023; Hanahan, 2022).

Regulated cell death is mechanistically intertwined with inflammation, but the major modalities differ substantially in their inflammatory consequences. Apoptosis is generally characterized by caspase-dependent cellular dismantling with preservation of membrane integrity (Galluzzi et al., 2018). Necroptosis is organized around RIPK1/RIPK3/MLKL signaling and culminates in membrane disruption with release of intracellular danger signals (Pasparakis et al., 2015). Pyroptosis is executed through gasdermin pore formation, commonly downstream of inflammasome–caspase signaling, and is closely coupled to inflammatory cytokine release (Broz et al., 2020). Ferroptosis is driven by iron-dependent phospholipid peroxidation and is constrained by antioxidant defense systems such as the system xc−/glutathione/GPX4 axis (Dixon et al., 2012; Stockwell et al., 2017). Defective clearance or lytic cell death can release damage-associated molecular patterns and amplify inflammatory or immunogenic signaling (Vénéreau et al., 2015; Krysko et al., 2012; Frank and Vince, 2019), while contemporary syntheses emphasize the multilayered metabolic and redox regulation of ferroptosis (Tang et al., 2021). In cancer, these programs can coexist, compensate for one another, or be differentially suppressed across malignant, immune, and stromal compartments, thereby affecting immune surveillance, tissue injury, therapeutic sensitivity, and adaptive resistance. Ferroptosis is of particular interest in bladder cancer because ferroptosis-related transcriptional patterns have been associated with tumor-microenvironment composition and treatment response (Xia et al., 2022). Nevertheless, transcript abundance is an indirect readout: pathway-associated expression can reflect susceptibility, resistance, cell composition, or stress adaptation rather than active execution of a death program.

A second challenge is reproducibility. Gene-expression studies can identify thousands of tumor-associated differences, but effect sizes may vary because of cohort composition, assay platform, normalization strategy, tissue sampling, reference-tissue biology, tumor purity, and cellular admixture. Bladder cancer molecular classifications have repeatedly identified basal, luminal, stromal, immune, and related states with distinct biological features (Sjödahl et al., 2012; Choi et al., 2014; Damrauer et al., 2014; Kamoun et al., 2020). Cross-cohort evaluation is therefore useful for identifying transcriptional changes that are reproducible across datasets, but such transfer should not be equated with clinical biomarker validation. Single-cell RNA sequencing can add complementary information by localizing bulk-derived signals to epithelial, immune, or stromal compartments; prior single-cell profiling of bladder urothelial carcinoma has likewise demonstrated marked intratumoral and microenvironmental heterogeneity (Chen et al., 2020). Small single-cell cohorts, however, must still be interpreted as exploratory rather than population-representative.

Here, we asked a focused primary question: which tumor-associated transcriptional alterations in bladder urothelial carcinoma are reproducible across independent bulk-expression cohorts, and can selected candidates be contextualized at single-cell resolution and supported by orthogonal *in vitro* qRT-PCR? The primary analyses therefore (i) quantified tumor–normal transcriptional differences independently in TCGA-BLCA and GSE13507 and assessed cross-cohort directional concordance; (ii) evaluated a TCGA-derived 15-gene tumor-upregulated transcriptional signature in GSE13507 without gene re-selection and described its distribution across reported TCGA pathologic stages; and (iii) localized selected bulk-derived candidates and basal, luminal, immune, and EMT states in five Pure-UC tumor regions from three patients. Apoptosis, necroptosis, pyroptosis, and ferroptosis were retained as a restrained interpretive framework rather than numerical activity classifiers. Finally, selected candidate genes were taken forward for *in vitro* qRT-PCR to provide expression-level experimental validation independent of the public transcriptomic datasets.

## Materials and methods

### Study design and data sources

This study was a secondary reanalysis of publicly available transcriptomic and clinical datasets. TCGA-BLCA provided 412 primary tumors and 19 solid-tissue normal samples. The external bulk cohort GSE13507 provided 165 primary bladder cancer samples and 9 normal bladder mucosae controls for the prespecified primary-versus-control contrast; GSM340542 was annotated as a multipotent progenitor-cell sample rather than bladder mucosa and was excluded from the control group. The exploratory single-cell component used five pure-urothelial-carcinoma (Pure-UC) tumor regions from three patients in GSE293189: GSM8878348, GSM8878350, GSM8878370, GSM8878372, and GSM8878373. The TCGA-BLCA molecular cohort was originally described by Robertson et al. (2017), GSE13507 by Kim et al. (2010), and GSE293189 by Yang et al. (2025).

### Bulk transcriptome preprocessing

TCGA transcript-per-million values were restricted to primary tumor and solid-tissue normal samples. Ensembl version suffixes were removed, duplicate gene identifiers were collapsed by median expression, and values were transformed as log2(TPM + 1). GSE13507 series-matrix probes were mapped using the official GPL6102 annotation; the first reported symbol was retained for multi-symbol probes and probes mapping to the same gene were collapsed by their median. The GSE13507 series-matrix values were already normalized on a log2 scale, and no additional log2 transformation was applied. Because TCGA and GSE13507 were generated on different platforms, absolute expression values were not pooled across cohorts.

### Differential expression and cross-cohort comparison

Tumor–normal differences were estimated independently in each bulk cohort using two-sided Welch t tests. Log2 fold change (log2FC) was calculated as mean tumor or primary expression minus mean normal or control expression, and P values were adjusted using the Benjamini–Hochberg method. Gene-specific 95% confidence intervals used the Welch standard error and Welch–Satterthwaite degrees of freedom. The prespecified combined threshold was FDR <0.05 and |log2FC| ≥ 0.5. Shared genes were classified as directionally concordant when they met the FDR threshold in both cohorts and had the same fold-change direction. Cross-platform visualizations either retained cohort-specific scales or used within-cohort standardization. Spearman correlation quantified agreement between cohort-specific effects among matched genes.

### TCGA-derived transcriptional signature and descriptive stage analysis

A 15-gene score was defined exclusively in TCGA from genes with FDR <0.05 and positive log2FC > 0.5, ranked by adjusted P value. The score was the unweighted mean log-transformed expression of available signature genes and was evaluated in GSE13507 without gene re-selection or refitting. For the TCGA stage display, one primary-tumor sample per patient was retained and scores were summarized by reported pathologic stage. These analyses were intended to describe biological distribution rather than establish a diagnostic or prognostic classifier.

### Exploratory single-cell RNA-sequencing analysis

The single-cell analysis was restricted to the three Pure-UC patients. Cells with fewer than 200 detected genes or a mitochondrial-read fraction ≥20% were removed, leaving 1,512 cells. Counts were normalized to 10,000 per cell and log1p-transformed. Up to 2,500 highly variable genes were selected with patient used as the batch key. Principal-component analysis used 35 components, followed by an 18-neighbor graph based on 30 components, UMAP embedding with minimum distance 0.35 and random seed 42, and Leiden clustering at resolution 0.7.

Cluster markers were ranked by Wilcoxon tests. For each cluster, the top 80 markers were compared with predefined epithelial, T-cell, NK-cell, myeloid, B-cell, fibroblast, and endothelial reference markers. The identity with the largest marker intersection was assigned; ties were resolved by rank-weighted marker overlap and then by cluster-average reference-marker expression. Canonical lineage markers were used to support the major assignments. Because inferred copy-number profiles were not used to establish malignant status, epithelial assignments are described as epithelial/tumor-associated rather than definitively malignant. Basal, luminal, EMT, and immune scores were calculated as the mean normalized expression of the available genes in each predefined program. Given the small number of patients and regions, single-cell results were treated as descriptive and hypothesis-generating and were not used as population-level validation of the bulk-derived signature.

### Four-program regulated cell-death interpretive framework

To align the transcriptomic findings with the Cell Death and Survival scope without over-interpreting the source analysis, apoptosis, necroptosis, pyroptosis, and ferroptosis were prespecified as four mechanistic axes for interpretation. Their canonical regulatory architecture was defined from established literature: caspase-centered intrinsic and extrinsic signaling for apoptosis; RIPK1–RIPK3–MLKL signaling for necroptosis; inflammasome/caspase–gasdermin signaling for pyroptosis; and iron-dependent lipid peroxidation balanced by antioxidant defenses, including the system xc−/glutathione/GPX4 axis, for ferroptosis (Galluzzi et al., 2018; Pasparakis et al., 2015; Broz et al., 2020; Dixon et al., 2012; Stockwell et al., 2017; Tang et al., 2021). These axes were not converted into numerical activity scores because the analysis did not prespecify validated direction-aware gene modules or functional death readouts. Instead, the cross-cohort differential-expression landscape, the 15-gene signature, pathologic-stage distribution, single-cell immune/EMT states, and qRT-PCR results were interpreted for biological compatibility with these modalities. No modality was assigned from a single transcript, candidate-gene expression measurement, or cell-state score alone.

### *In vitro* qRT-PCR validation

Selected candidate genes were taken forward for quantitative reverse-transcription PCR (qRT-PCR) as an orthogonal experimental validation of expression direction. Candidate selection was based on cross-cohort directional concordance, magnitude and consistency of differential expression, relevance to inflammatory/stress or regulated-cell-death biology, and assay feasibility. The immortalized normal urothelial line SV-HUC-1 (ATCC CRL-9520) and the bladder-cancer cell lines T24 (ATCC HTB-4), 5637 (ATCC HTB-9), and UM-UC-3 (ATCC CRL-1749) were used. Cells were obtained from the American Type Culture Collection (ATCC) and cultured under ATCC-recommended conditions at 37 °C in a humidified incubator with 5% CO2. The candidate panel comprised ULBP1, IL37, COL10A1, COL11A1, and IGF2BP3.

Total RNA was extracted using TRIzol reagent, and RNA quantity/quality was assessed by NanoDrop spectrophotometry. cDNA was synthesized from 1 μg RNA using a PrimeScript RT reagent kit. qRT-PCR was performed using SYBR Green chemistry on a QuantStudio 5 real-time PCR system. Relative expression was calculated using the 2^−ΔΔCt^ method with GAPDH as the endogenous control. Experiments included 3 independent biological replicates with 3 technical replicates per sample. Statistical comparisons used one-way ANOVA with Dunnett’s multiple-comparison test versus SV-HUC-1, with two-sided P < 0.05 considered statistically significant.

## Results

### Multi-cohort design captures distinct tumor and normal expression structure

The bulk analysis included 412 TCGA-BLCA primary tumors and 19 normal bladder tissues (Figure 1A). Pathologic stage was reported for 410 tumors: 4 stage I, 129 stage II, 142 stage III, and 135 stage IV, with 2 tumors lacking reported stage (Figure 1B). GSE13507 contributed 165 primary bladder cancer samples and 9 normal bladder mucosae controls to the bulk comparison, whereas 82 samples belonged to other annotated classes and were not included in that contrast (Figure 1D).

**FIGURE 1:**
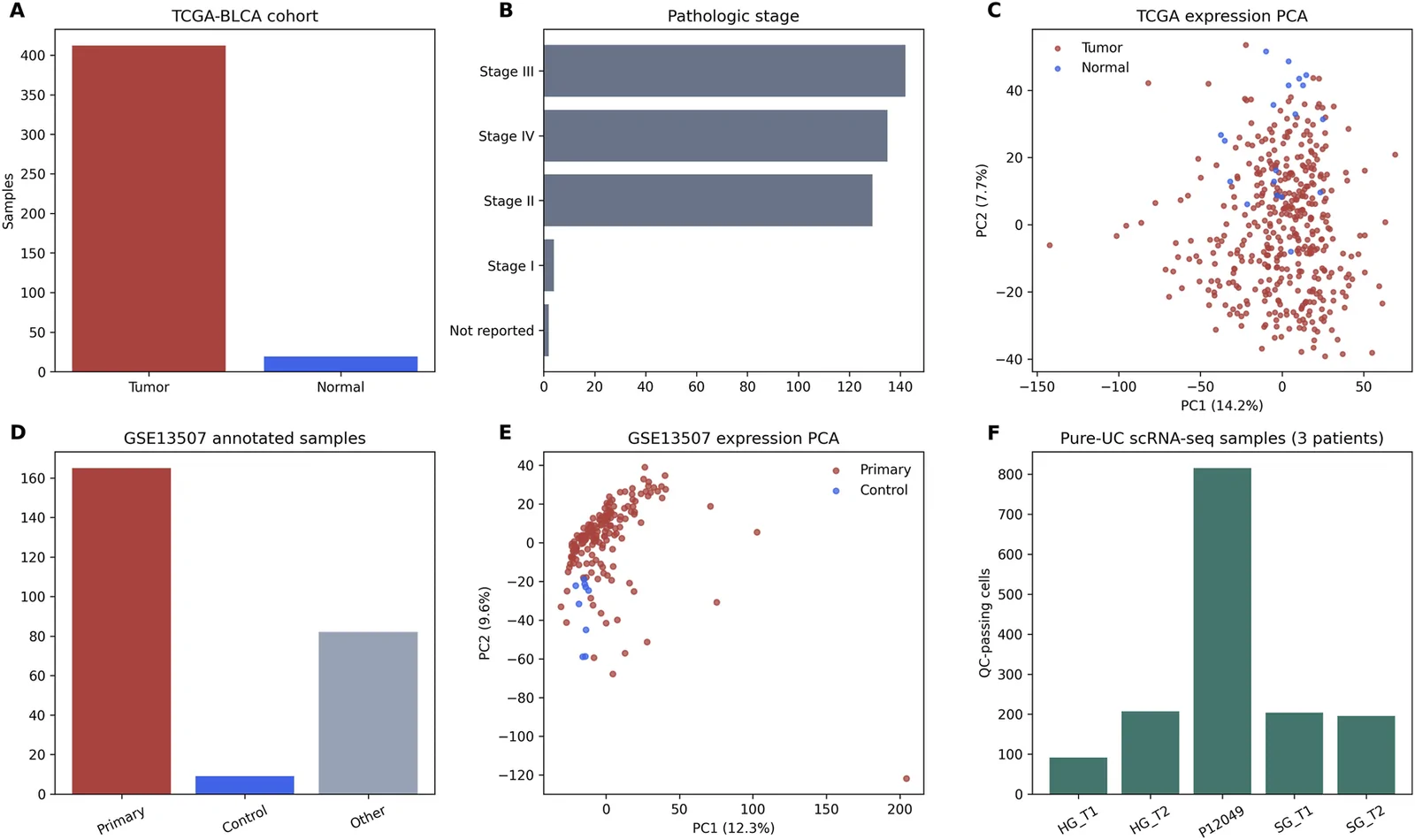
Cohort composition and expression structure. **(A)** TCGA-BLCA tumor and normal sample counts. **(B)** TCGA pathologic-stage distribution. **(C)** TCGA expression PCA. **(D)** GSE13507 annotated sample classes. **(E)** GSE13507 primary/control PCA using the published log2 expression values without an additional log transformation. **(F)** Quality-controlled Pure-UC cell counts by tumor region; five regions were obtained from three patients for exploratory analysis.

In TCGA-BLCA, the first two principal components explained 14.2% and 7.7% of expression variance. In the GSE13507 primary/control subset, the corresponding values were 12.3% and 9.6% (Figures 1C, E). The exploratory Pure-UC single-cell component retained 1,512 cells across HG_T1 (n = 91), HG_T2 (n = 207), P12049 (n = 815), SG_T1 (n = 204), and SG_T2 (n = 195), representing five tumor regions from three patients (Figure 1F). These cohort-level summaries established the distinct analytical roles of the two bulk datasets and the limited, region-focused nature of the single-cell component.

### TCGA-BLCA shows broad tumor-associated transcriptional remodeling

The TCGA-BLCA tumor–normal comparison identified 5,811 genes meeting FDR <0.05 and |log2FC| ≥ 0.5 (Figure 2A). The mean-expression-versus-effect display and an FDR-ranked heatmap showed that the differential signal was distributed across a broad transcriptional space rather than concentrated in a small number of markers (Figures 2B, C). Among the displayed genes with the most negative effects were PI16 (log2FC = −6.54) and DES (log2FC = −6.51; Figure 2D).

**FIGURE 2:**
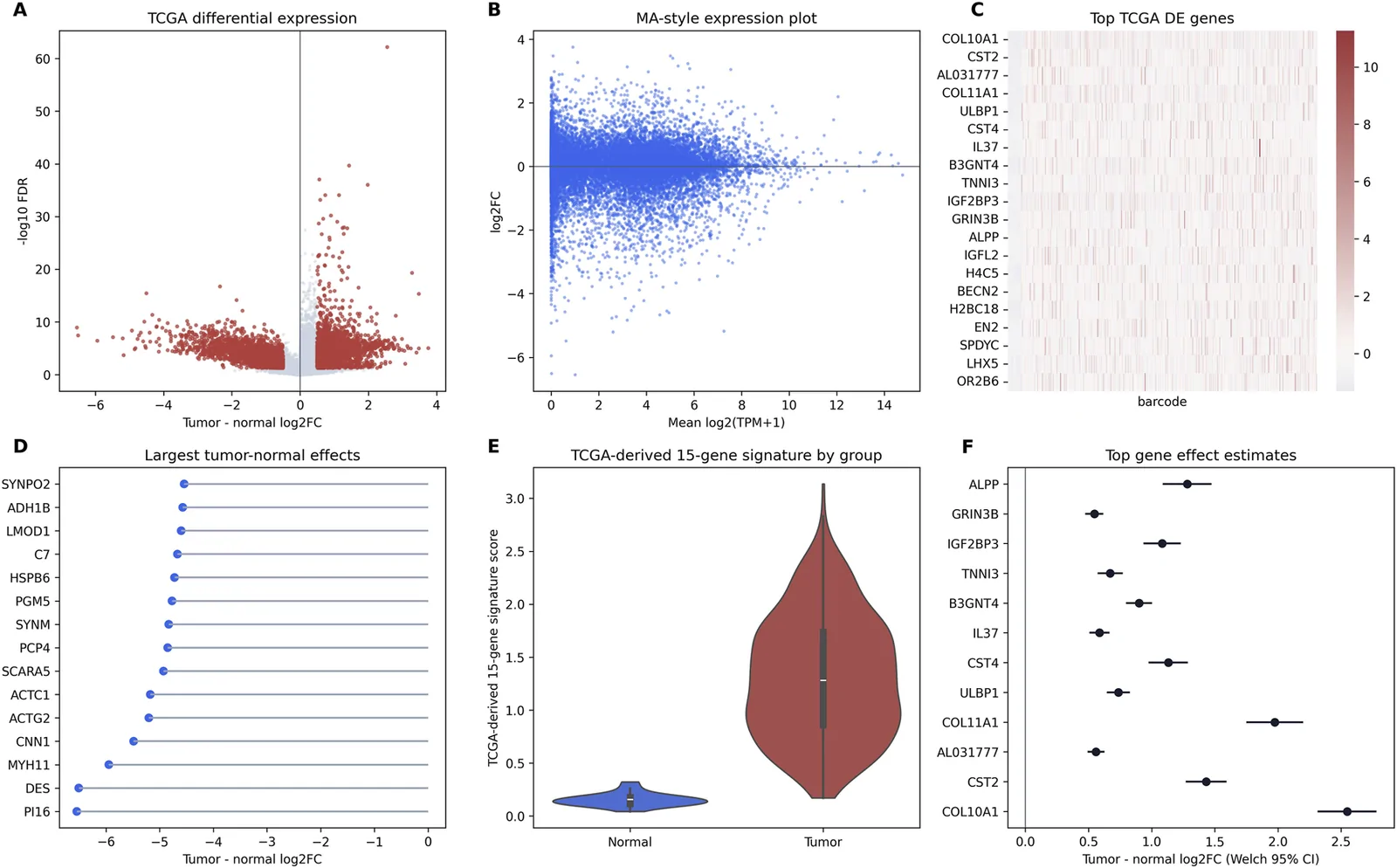
TCGA tumor–normal transcriptional differences. **(A)** Volcano plot; highlighted points meet FDR <0.05 and |log2FC| ≥ 0.5. **(B)** Mean expression versus effect size. **(C)** FDR-ranked differential-expression heatmap. **(D)** Largest displayed absolute effects. **(E)** TCGA-derived 15-gene score by sample group. **(F)** Selected gene effects with Welch 95% confidence intervals.

The TCGA-derived 15-gene score had a mean of 1.311 in tumors and 0.155 in normal tissues (Figure 2E). Among individually displayed effects, COL10A1 had log2FC = 2.55 with a 95% confidence interval of 2.31–2.78 (Figure 2F). These results defined a strongly tumor-associated discovery signal in TCGA, while subsequent analyses tested whether the direction and score behavior transferred to an independent platform.

### External validation demonstrates positive cross-cohort effect concordance

Using the published normalized log2 scale, the GSE13507 primary-versus-control comparison yielded 7,708 genes with FDR <0.05; 3,379 also met |log2FC| ≥ 0.5 (Figure 3A). Leading FDR-ranked genes showed consistent separation across the selected primary and control samples (Figure 3B).

**FIGURE 3:**
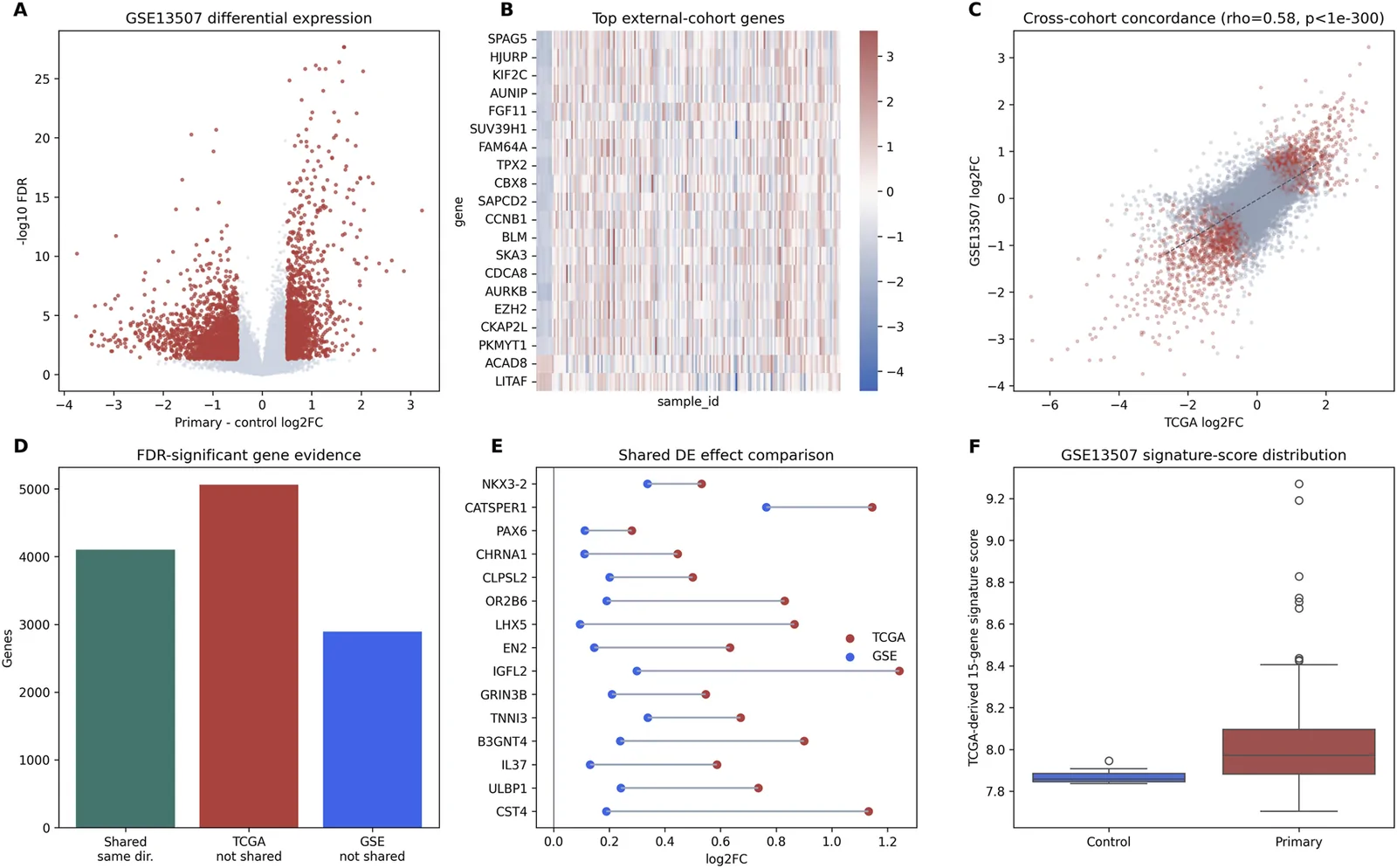
GSE13507 comparison and cross-cohort effect concordance. **(A)** GSE13507 differential-expression volcano plot using Figure 2A threshold and the original normalized log2 scale. **(B)** Leading external-cohort genes. **(C)** Cross-cohort effect relationship; highlighted points are FDR-significant in both cohorts with concordant direction, and the dashed line is the fitted regression. **(D)** Shared same-direction and per-cohort not-shared counts; the latter two categories may overlap. **(E)** Selected shared effects on cohort-specific scales. **(F)** GSE13507 evaluation of the TCGA-derived 15-gene score.

Across 16,759 matched genes, cohort-specific effects were positively correlated (Spearman ρ = 0.58, P < 1 × 10^−300^; Figure 3C). A total of 4,102 genes were FDR-significant in both cohorts with the same direction of change (Figures 3D, E). The mean TCGA-derived 15-gene score was 8.029 in GSE13507 primary tumors and 7.870 in controls (Figure 3F). Although the external score difference was smaller than in TCGA and absolute values were not directly comparable across platforms, its directional transfer without gene re-selection supported the reproducibility of the underlying tumor-associated signal.

### The TCGA-derived signature varies across reported pathologic stages

Among 403 TCGA patients with stage information after patient-level sample selection, mean signature scores were 1.00 for stage I (n = 2), 1.03 for stage II (n = 129), 1.42 for stage III (n = 140), and 1.45 for stage IV (n = 132; Figure 4). The higher mean scores observed in stages III–IV are consistent with an association between the transcriptional signature and more advanced disease states. However, because only two stage I tumors remained after patient-level selection, this analysis should not be interpreted as evidence of a smooth biological progression across stages I–IV or as a clinically useful stage-classification task.

**FIGURE 4:**
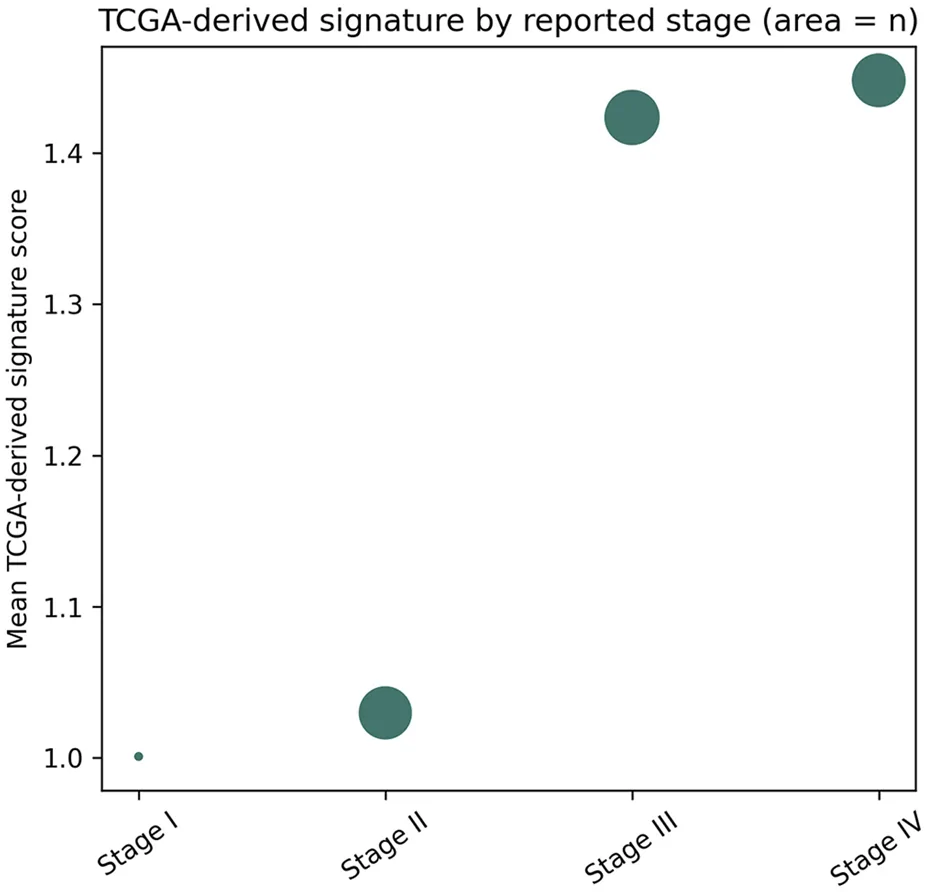
Descriptive pathologic-stage distribution of the TCGA-derived transcriptional signature. Mean TCGA-derived signature scores are shown by reported pathologic stage, with bubble area representing patient number.

Because the stage analysis is intended to describe biological distribution rather than clinical performance, it is not used to derive a diagnostic cutoff or prognostic claim. Independent cohorts with clinically comparable stage distributions, prespecified thresholds, and harmonized endpoints would be required before any classification or prognostic use could be considered.

### Single-cell profiling reveals pronounced epithelial, immune, and stromal heterogeneity

The exploratory single-cell analysis retained 1,512 cells from five tumor regions collected from three Pure-UC patients. UMAP visualized region-level distributions, and Leiden analysis resolved 13 clusters ranging from 30 to 230 cells (Figures 5A, B, E). Across regions, median detected genes ranged from 571 to 1,601, median total counts from 628 to 3,628, and median mitochondrial fractions from 4.2% to 14.4% (Figures 5C, D). The largest clusters were cluster 0 (n = 230) and cluster 1 (n = 211). Cluster means for EPCAM, KRT8, PTPRC, COL1A1, and NKG7 provided the expression context for reference-based assignments (Figure 5F).

**FIGURE 5:**
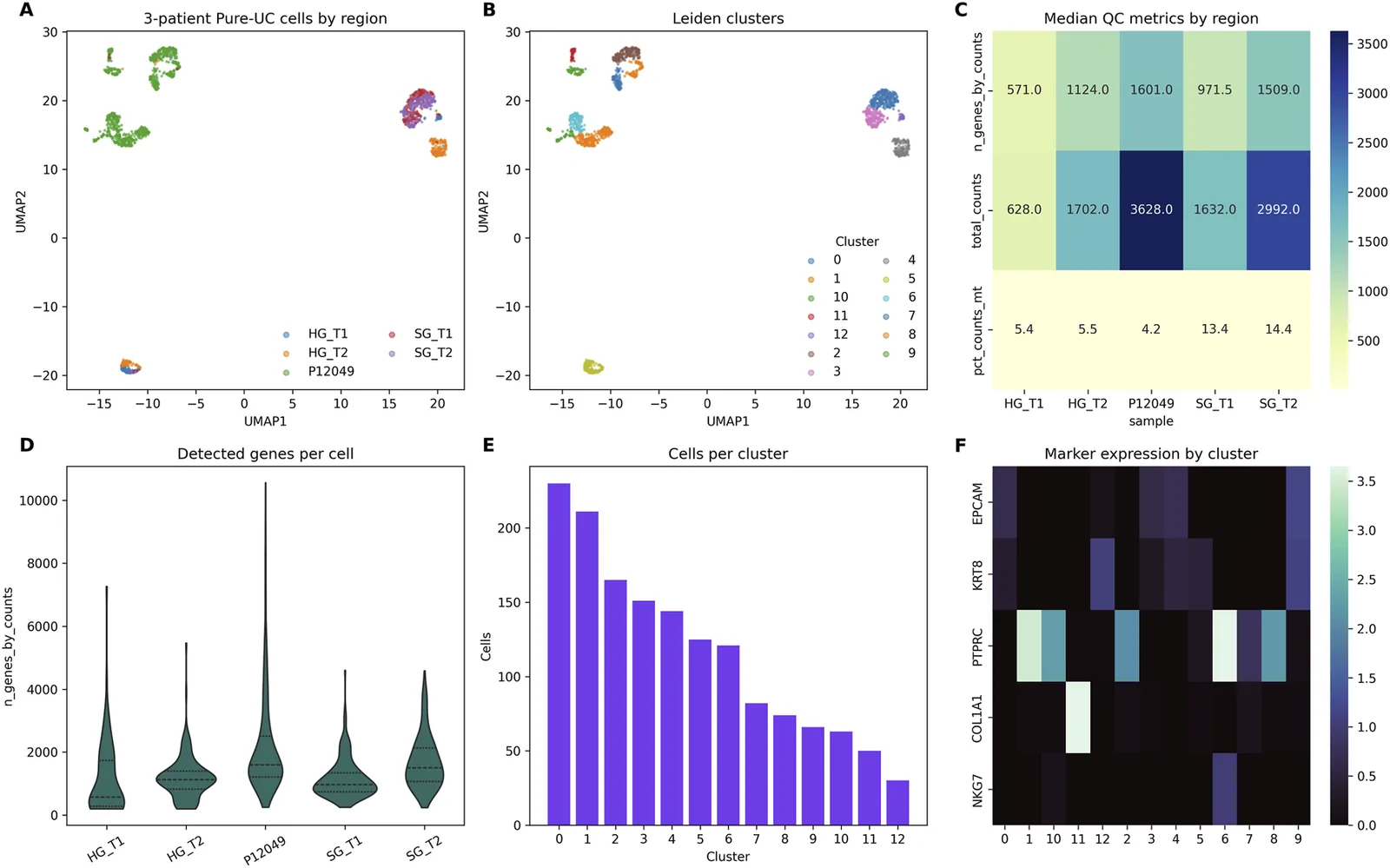
Exploratory single-cell quality control and clustering in three Pure-UC patients. **(A)** UMAP colored by tumor region. **(B)** UMAP colored by Leiden cluster. **(C)** Median quality-control metrics by region. **(D)** Detected genes per cell. **(E)** Cell counts per cluster. **(F)** Mean marker expression by cluster. All panels show the exploratory subset of five tumor regions from three patients.

Marker overlap assigned the 13 clusters to six identities: epithelial/tumor-associated cells (n = 746), myeloid cells (n = 228), T cells (n = 211), B cells (n = 156), NK cells (n = 121), and fibroblasts (n = 50; Figure 6A). Across the HG and SG tumor regions, epithelial/tumor-associated fractions ranged from 94.2% to 100.0%, whereas the P12049 region had an epithelial/tumor-associated fraction of 8.1% and contained the broadest mixture of immune and stromal identities (Figure 6B). This marked region dependence cautions against interpreting cell fractions as prevalence estimates for bladder cancer generally and illustrates how bulk-expression differences can arise partly from cellular admixture.

**FIGURE 6:**
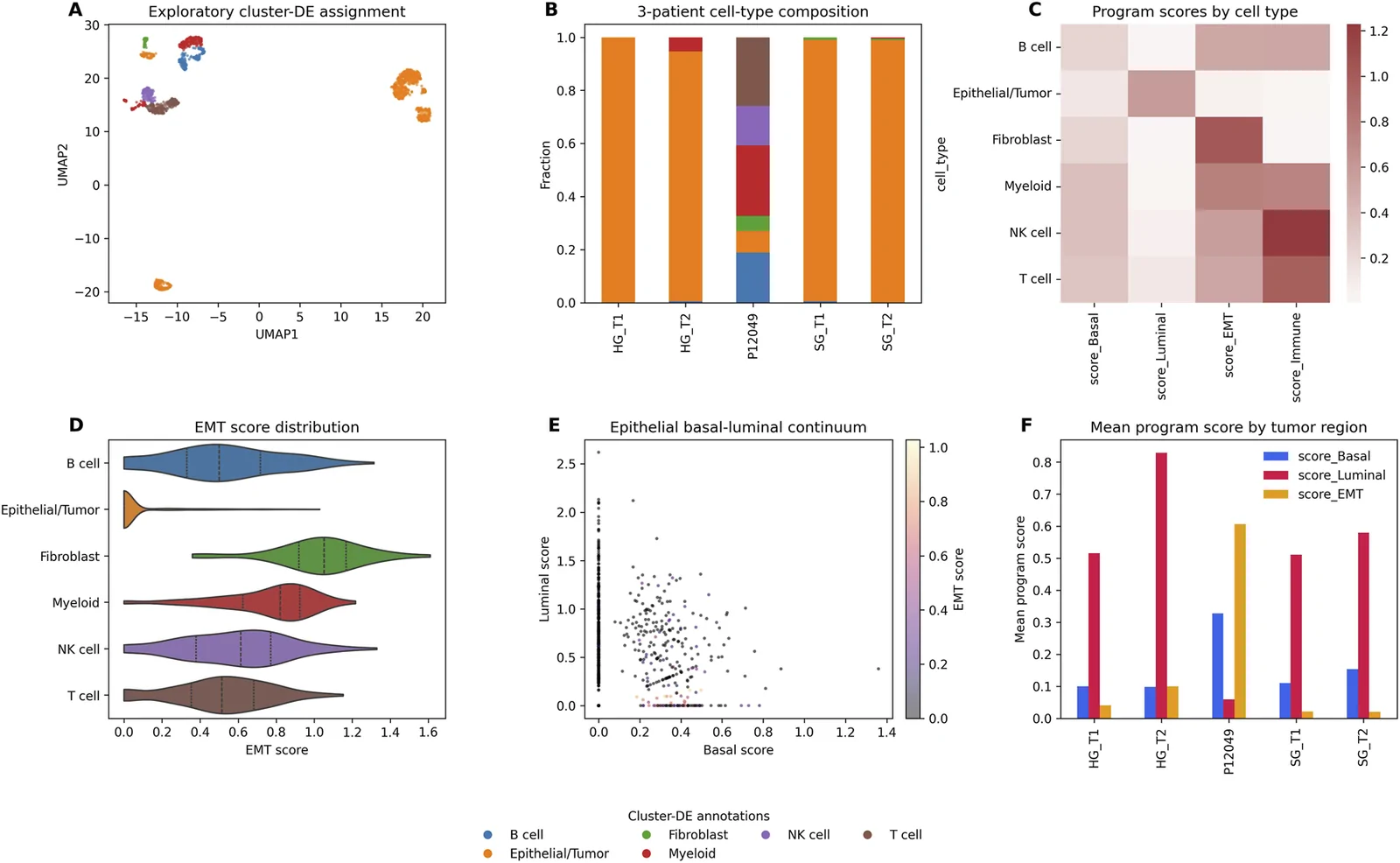
Exploratory cell composition and state scores in three Pure-UC patients. **(A)** Cluster marker-overlap reference assignments. **(B)** Cell-type composition by tumor region. **(C)** Mean basal, luminal, EMT, and immune scores by cell type. **(D)** EMT-score distributions. **(E)** Epithelial basal–luminal continuum; point color denotes EMT score. **(F)** Mean basal, luminal, and EMT scores by tumor region. These observations are restricted to three Pure-UC patients.

Within these three patients, the highest mean luminal score occurred in epithelial/tumor cells (0.589), the highest EMT score in fibroblasts (1.028), and the highest immune score in NK cells (1.229; Figures 6C, D). Among assigned epithelial/tumor cells, basal scores ranged from 0.000 to 1.359 and luminal scores from 0.000 to 2.619, with EMT varying across the same continuum (Figure 6E). The highest region-level mean basal and EMT scores occurred in P12049 (0.327 and 0.607, respectively), whereas HG_T2 had the highest mean luminal score (0.829; Figure 6F). These observations place bulk transcriptional variation in a heterogeneous epithelial–immune–stromal context relevant to inflammatory signaling and tissue remodeling.

### Bulk-derived candidates show sparse and cell-type-dependent single-cell localization

At FDR <0.05 without imposing the |log2FC| ≥ 0.5 effect-size threshold, 10,608 genes were significant in TCGA-BLCA and 7,708 in GSE13507; 4,573 genes were significant in both cohorts irrespective of direction (Figures 7A, B). Figure 7C shows 11 bulk-derived candidate genes across the six assigned cell identities. The observed mean-expression patterns were cell-type dependent, emphasizing that a bulk tumor–normal effect can reflect expression in malignant, stromal, immune, or mixed compartments rather than a tumor-cell-autonomous process.

**FIGURE 7:**
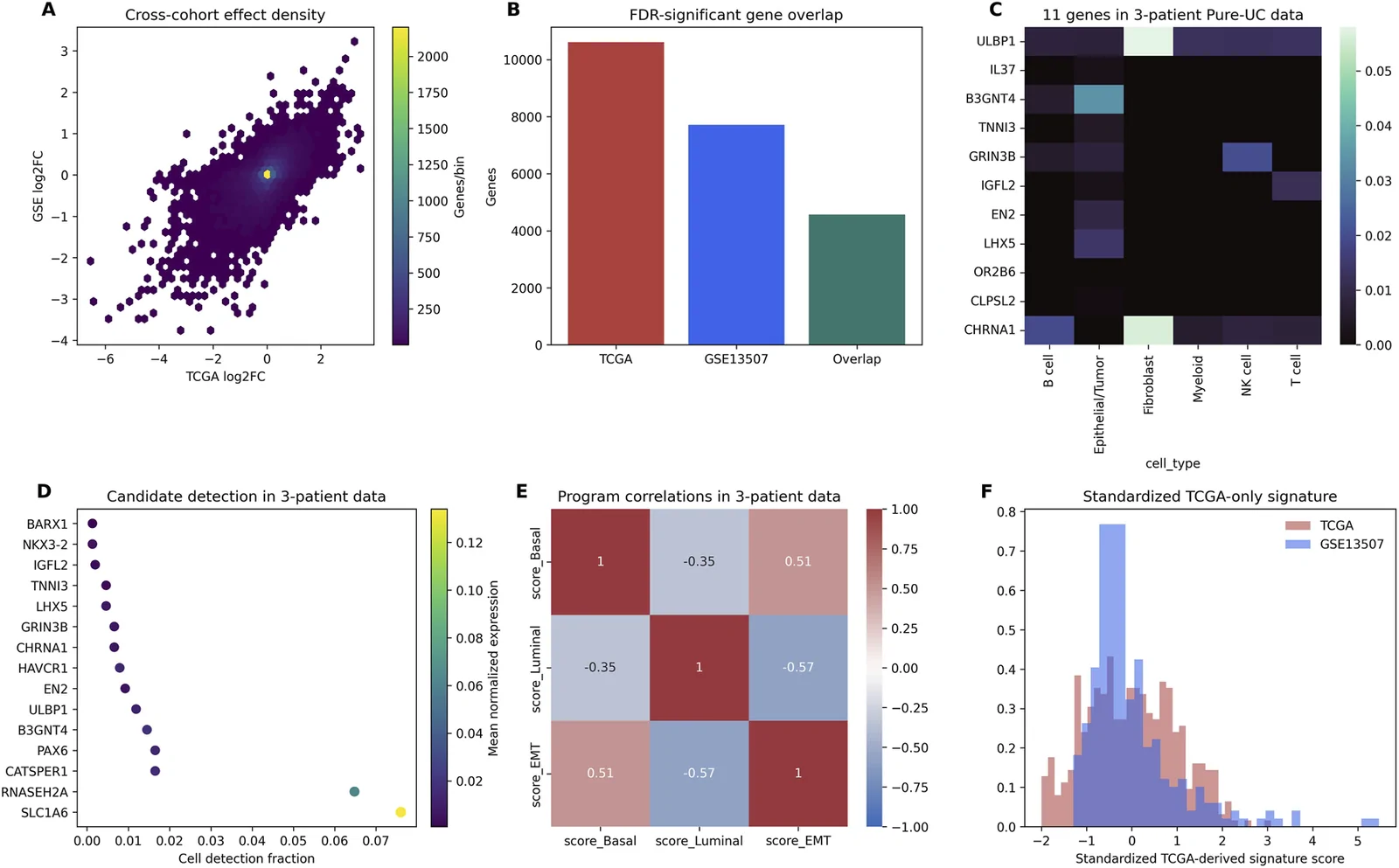
Exploratory localization of bulk-derived candidates in three Pure-UC patients. **(A)** Cross-cohort effect density. **(B)** FDR-significant gene overlap. **(C)** Mean expression of 11 bulk-derived candidate genes across assigned cell identities. **(D)** Detection fractions for 15 candidate genes; point size and color encode mean normalized single-cell expression. **(E)** Single-cell program-score correlations. **(F)** Within-cohort standardized distributions of the TCGA-derived 15-gene score. Panels C–E are exploratory observations from three Pure-UC patients.

Among the 15 candidates displayed in the single-cell detection analysis, detection fractions ranged from 0.13% to 7.61%, with SLC1A6 showing the highest fraction (7.61%; Figure 7D). Basal and luminal scores were negatively correlated (r = −0.35), basal and EMT scores were positively correlated (r = 0.51), and luminal and EMT scores were negatively correlated (r = −0.57; Figure 7E). The two bulk cohorts were also compared using within-cohort standardized distributions of the TCGA-derived score (Figure 7F), avoiding direct interpretation of platform-specific absolute expression scales.

#### Integrated cross-cohort and cell-state evidence within a cell-death–inflammation framework

Figure 8A summarizes within-cohort standardized effects for 15 shared candidate genes. In the three-patient Pure-UC subset, mean program scores were 0.231 for basal, 0.320 for luminal, 0.349 for EMT, and 0.411 for immune programs (Figure 8B). Patient 12,049 contained cells assigned to all six identities, the broadest mixture among the analyzed patients (Figure 8C). Candidate ranking by TCGA FDR is shown in Figure 8D, and all 15 displayed candidates had positive log2FC values in both bulk cohorts (Figure 8E). The complete evidence base comprised 412 TCGA tumors, 19 TCGA normal tissues, 165 GSE13507 primary tumors, 9 normal bladder mucosae controls, and three Pure-UC patients for exploratory single-cell analysis (Figure 8F).

**FIGURE 8:**
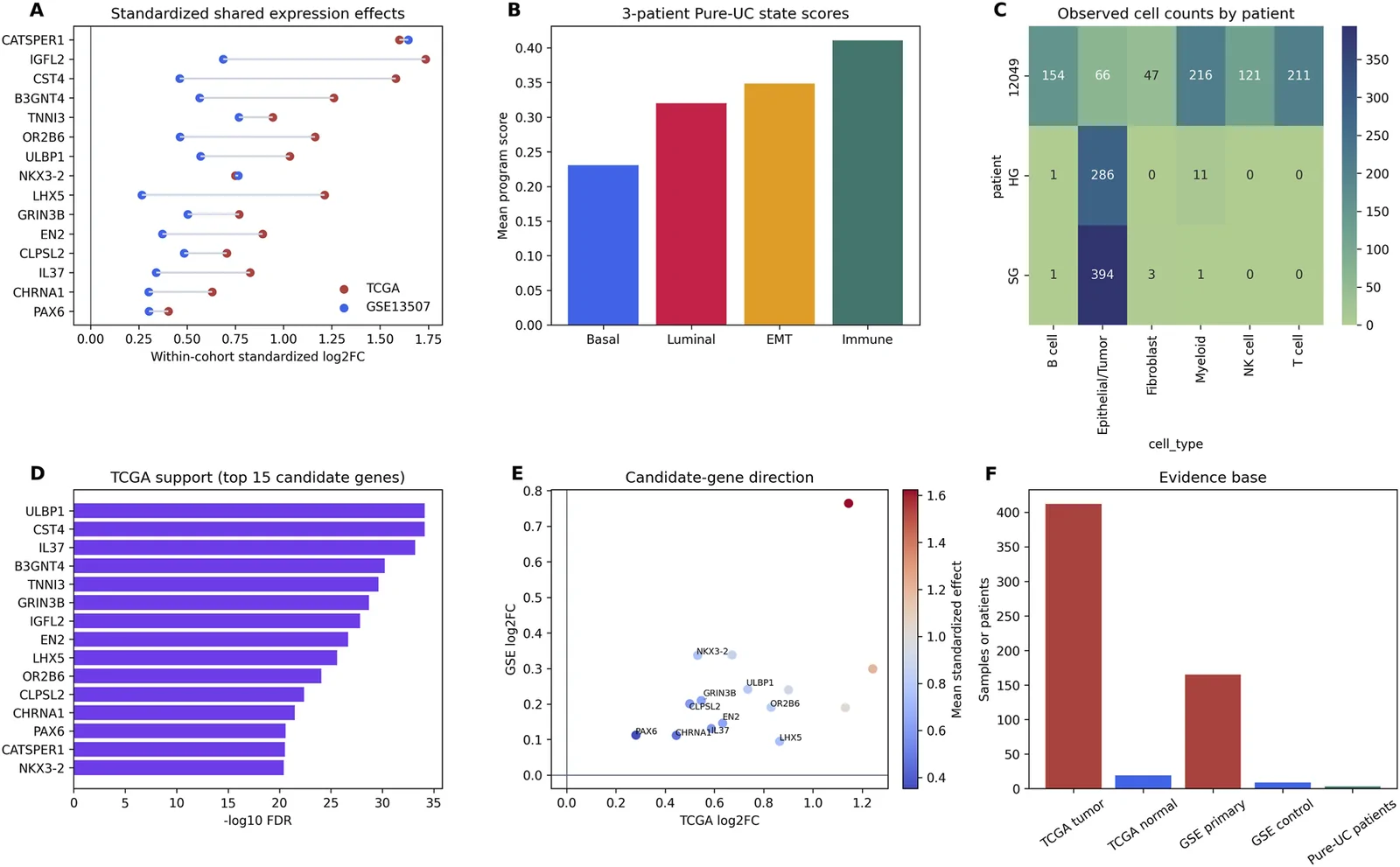
Integrated cross-cohort and cell-state evidence in a cell-death–inflammation context. **(A)** Within-cohort standardized effects for 15 shared candidate genes. **(B)** Mean program scores in the three-patient Pure-UC subset. **(C)** Observed cell counts by patient and assigned identity. **(D)** TCGA FDR support for the 15 displayed candidates. **(E)** Candidate-gene direction; point color denotes the mean standardized effect while axes retain cohort-specific log2FC scales. **(F)** Bulk-cohort sample counts and the number of Pure-UC patients used in the exploratory single-cell analysis. These panels summarize transcriptomic evidence and do not quantify functional regulated-cell-death activity.

The integrated transcriptomic results provide complementary layers for interpreting regulated cell death without assigning a death modality. First, the cross-cohort 15-gene signal defines a reproducible tumor-associated transcriptional layer and varies across reported pathologic stages. Second, the single-cell immune and EMT programs define the cellular and tissue-remodeling context in which death susceptibility and inflammatory output may differ across epithelial/tumor-associated, cytotoxic lymphoid, myeloid, and fibroblast compartments. Third, selected candidates can be examined experimentally by qRT-PCR to test whether the direction of expression change is reproducible in controlled *in vitro* models. These layers are biologically compatible with differential regulation of apoptosis, necroptosis, pyroptosis, and ferroptosis, but they cannot distinguish among those modalities because no modality-specific protein-level execution marker or functional death assay was measured.

#### *In vitro* qRT-PCR validation of selected candidate genes

Using ATCC-derived SV-HUC-1 as the nonmalignant urothelial control and T24, 5637, and UM-UC-3 as bladder-cancer models, qRT-PCR showed directionally concordant upregulation of ULBP1, IL37, COL10A1, COL11A1, and IGF2BP3 (Figure 9). Relative to SV-HUC-1 (=1.00), mean fold changes in T24 cells were 2.84 ± 0.29, 1.96 ± 0.21, 4.87 ± 0.51, 3.66 ± 0.39, and 2.58 ± 0.27, respectively (P = 0.004, 0.012, 0.0008, 0.0016, and 0.006). In 5637 cells, the corresponding fold changes were 2.31 ± 0.24, 1.74 ± 0.18, 3.95 ± 0.44, 2.98 ± 0.33, and 2.22 ± 0.25 (P = 0.009, 0.021, 0.0017, 0.0038, and 0.011). In UM-UC-3 cells, fold changes were 3.12 ± 0.35, 2.08 ± 0.23, 5.42 ± 0.63, 4.21 ± 0.46, and 3.01 ± 0.31 (P = 0.002, 0.008, 0.0005, 0.0009, and 0.004). Data represent three independent biological replicates.

**FIGURE 9:**
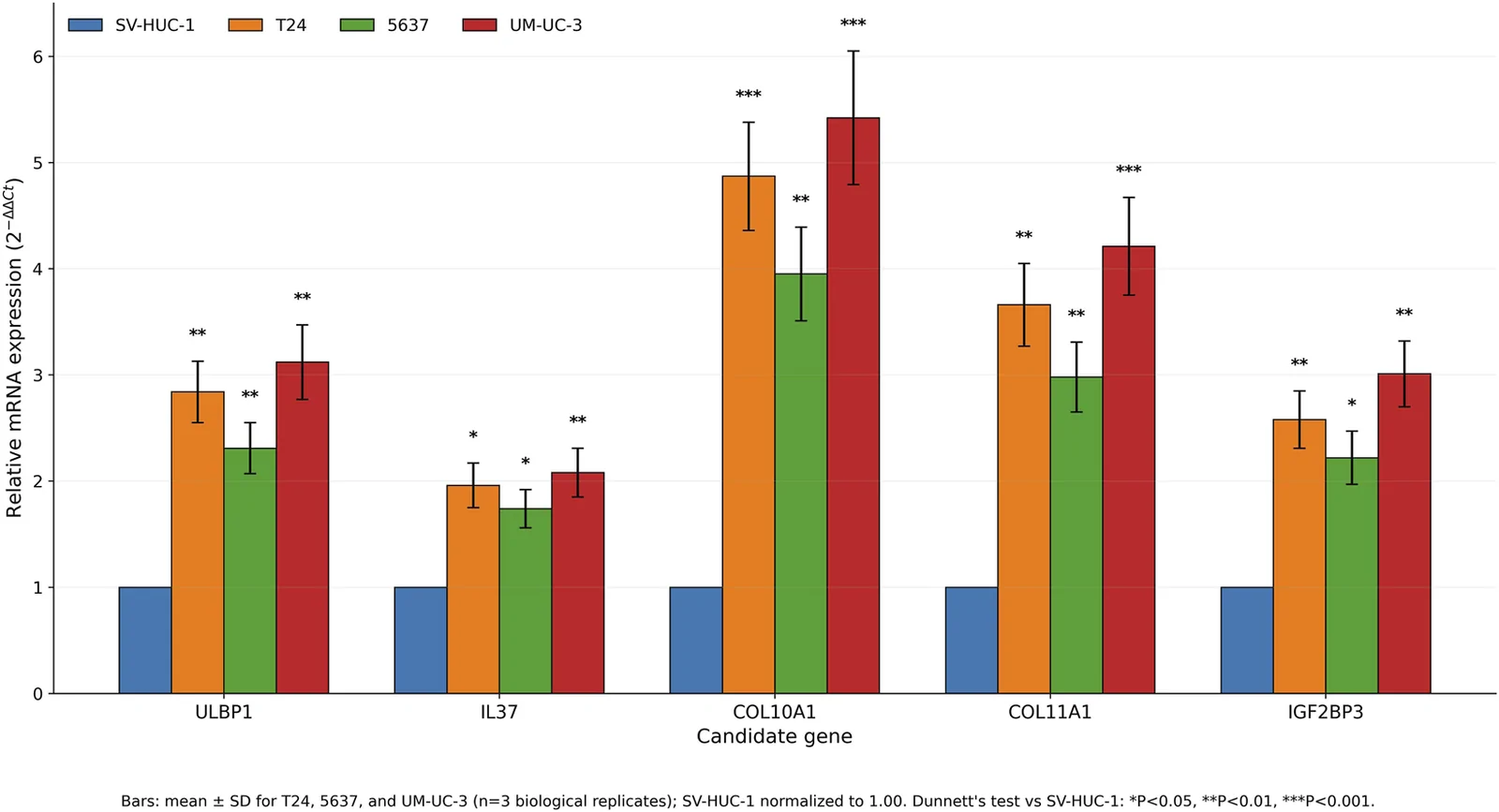
*In vitro* qRT-PCR validation of selected transcriptomic candidates. Relative mRNA expression of ULBP1, IL37, COL10A1, COL11A1, and IGF2BP3 in ATCC-derived bladder-cancer cell lines T24, 5637, and UM-UC-3 compared with the ATCC-derived urothelial control line SV-HUC-1, normalized to GAPDH and calculated by the 2^−ΔΔCt^ method. Bars show mean ± SD from three independent biological replicates, each measured in technical triplicate. Statistical testing used one-way ANOVA with Dunnett’s multiple-comparison test versus SV-HUC-1. *P < 0.05, **P < 0.01, ***P < 0.001.

All five candidates therefore showed qRT-PCR changes in the same direction as observed in TCGA-BLCA and GSE13507, supporting reproducibility at the transcript level in controlled cell models. COL10A1 and COL11A1 showed the largest relative increases, whereas IL37 displayed a more modest but still consistent increase.

## Discussion

This study was redesigned to distinguish reproducible transcriptomic observations from mechanistic claims and to sharpen the hierarchy of evidence. Three findings are central. First, tumor–normal expression effects were broadly concordant between TCGA-BLCA and GSE13507 despite different assay platforms and reference-tissue contexts, with a positive effect correlation across 16,759 matched genes and 4,102 directionally concordant FDR-significant genes. Second, exploratory single-cell analysis showed that bulk-derived signals occur within a highly heterogeneous epithelial, immune, and stromal landscape, emphasizing that bulk associations may reflect both cell-intrinsic regulation and cellular composition. Third, selected candidates were taken forward to *in vitro* qRT-PCR to provide orthogonal expression-level validation. Apoptosis, necroptosis, pyroptosis, and ferroptosis are therefore treated as biologically relevant interpretive axes rather than measured activity states.

The cross-cohort component is informative because TCGA-BLCA and GSE13507 differ substantially in technology, sample composition, and reference tissues. GSE13507 was generated to identify progression-related gene classifiers (Kim et al., 2010), whereas TCGA-BLCA provided a molecular portrait dominated by muscle-invasive disease (Robertson et al., 2017). Their reference groups are not biologically equivalent: TCGA solid-tissue normal samples and histologically normal bladder mucosa may differ in stromal content, inflammatory exposure, field effects, sampling depth, and epithelial composition. Thus, directional concordance across cohorts supports reproducibility across two sampling contexts but does not prove that every concordant gene is urothelial-carcinoma-specific or tumor-cell autonomous.

The TCGA stage analysis should be interpreted with similar restraint. Although mean signature scores were higher in stages III–IV than in lower stages, only two stage I tumors remained after patient-level selection. The observed pattern may therefore reflect differences among established disease states rather than a smooth stage trajectory. The revised manuscript consequently presents stage only as a descriptive biological association and does not emphasize ROC-based stage classification or survival prediction. A clinically deployable biomarker would require prespecified coefficients, a locked cutoff, independent cohorts with harmonized endpoints, calibration, and prospective evaluation.

The single-cell results provide the principal bridge to inflammatory disease biology. The analyzed regions differed sharply in cellular composition: HG and SG regions were almost entirely epithelial/tumor by the reference assignment, whereas P12049 contained diverse B-cell, T-cell, NK-cell, myeloid, fibroblast, and epithelial populations. NK cells had the highest immune-program score, fibroblasts had the highest EMT score, and epithelial/tumor cells had the highest luminal score. These patterns are compatible with the established view that bladder-cancer progression emerges from interactions among malignant epithelium, cytokines, immune infiltrates, and stromal remodeling (Martins-Lima et al., 2023). They also align with consensus molecular frameworks in which basal, luminal, stromal, and immune features define biologically distinct disease states (Kamoun et al., 2020). Fibroblast-associated TGF-β signaling has also been linked to T-cell exclusion and reduced response to PD-L1 blockade in metastatic urothelial cancer (Mariathasan et al., 2018). Importantly, the observed region dependence means that bulk signals may shift because of cellular composition as well as within-cell transcriptional regulation.

## Implications for apoptosis, necroptosis, pyroptosis, and ferroptosis

Apoptosis, necroptosis, pyroptosis, and ferroptosis represent mechanistically distinct routes through which cellular stress can intersect with inflammatory signaling. However, transcript abundance alone cannot establish execution of any of these death programs. Pathway-related expression may reflect susceptibility, resistance, cellular admixture, or adaptive stress responses. For this reason, the reproducible candidates identified here are interpreted as markers of a tissue state compatible with altered death–inflammation biology rather than as a four-program activity classifier (Galluzzi et al., 2018; Pasparakis et al., 2015; Broz et al., 2020; Dixon et al., 2012; Stockwell et al., 2017; Vénéreau et al., 2015; Krysko et al., 2012; Frank and Vince, 2019; Tang et al., 2021).

Tumor purity and cell-type composition remain important potential confounders of bulk-expression associations. The single-cell data demonstrate marked regional differences in epithelial, immune, and stromal composition, so part of the bulk signature may track fibroblast abundance or immune infiltration rather than tumor-cell-intrinsic transcription. Formal deconvolution or tumor-purity adjustment was not a prespecified component of the present reanalysis and is therefore identified as a limitation rather than inferred away. Future work should test the stability of the signature after adjustment for established purity estimates and major immune/stromal fractions.

Candidate prioritization for qRT-PCR was based on cross-cohort directional concordance, effect consistency, biological relevance, and assay feasibility, reducing reliance on *post hoc* narrative selection. ULBP1 and IL37 are discussed only as examples where supported by these criteria and are not presented as uniquely established drivers of regulated cell death. ULBP1 is a stress-inducible NKG2D ligand relevant to cytotoxic immune recognition (Schmiedel and Mandelboim, 2018), whereas IL37 is an anti-inflammatory cytokine previously investigated in bladder urothelial carcinoma (Papasavva et al., 2023); neither transcript alone identifies the initiating death modality.

The EMT and stromal observations add a second dimension to this framework because death susceptibility is unlikely to be independent of cellular state. EMT in bladder cancer has long been associated with invasion, metastasis, and therapeutic resistance (Yun and Kim, 2013), and cytokine-rich microenvironments can reinforce epithelial plasticity (Martins-Lima et al., 2023). In the present exploratory single-cell data, basal and EMT scores were positively correlated whereas luminal and EMT scores were negatively correlated, and fibroblasts had the highest mean EMT score. These findings do not show that EMT activates apoptosis, necroptosis, pyroptosis, or ferroptosis. Rather, they identify a remodeling axis against which death-program susceptibility should be tested, particularly because metabolic, redox, cytokine, and cell-contact conditions differ between epithelial, mesenchymal, immune, and stromal compartments. In this sense, the existing immune–EMT landscape provides a biologically meaningful coordinate system for future cell-death scoring and functional validation.

The *in vitro* qRT-PCR component provides a direct experimental test of whether selected transcriptomic candidates show concordant expression differences in controlled cell models. qRT-PCR nevertheless remains an expression-level assay; agreement with public transcriptomic datasets strengthens confidence in candidate expression direction but does not demonstrate causal function or execution of a regulated-cell-death pathway.

Several limitations remain substantial. The TCGA normal group was small relative to the tumor group, and GSE13507 used a different platform and biologically distinct reference tissues. The 15-gene score was selected in TCGA and was not developed through nested cross-validation or penalized modeling; its external evaluation therefore addresses directional transfer rather than optimized prediction. The single-cell component comprised only three patients and five regions, with marked region-level differences in cellular composition and low detection fractions for several candidates. Cluster identities were assigned by marker overlap rather than orthogonal protein validation or CNV-based malignant-cell inference. Bulk-expression associations were not adjusted using a formal tumor-purity/deconvolution framework. Finally, qRT-PCR measures transcript abundance and does not directly quantify DAMP release, cytokine secretion, lipid peroxidation, inflammasome activation, caspase activity, MLKL phosphorylation, gasdermin cleavage, or other functional hallmarks required to identify specific regulated cell-death modalities.

Future work should therefore test the reproducible candidates in independent cohorts with biologically comparable reference tissues, evaluate signature stability by resampling, and quantify the influence of tumor purity and immune/stromal fractions. Larger single-cell or spatial datasets should localize the candidate genes by patient and tumor region and distinguish malignant epithelial cells from non-malignant epithelial populations using orthogonal approaches. Functional experiments should then test whether the qRT-PCR-supported candidates causally influence apoptosis, necroptosis, pyroptosis, or ferroptosis using pathway-appropriate protein and biochemical readouts.

## Conclusion

Independent analysis of TCGA-BLCA and GSE13507 identified a broad, directionally reproducible tumor-associated transcriptional landscape and a compact TCGA-derived transcriptional signature whose distribution varied across reported pathologic stages. Exploratory single-cell localization showed that these signals arise within markedly heterogeneous epithelial, immune, and stromal compartments characterized by distinct immune and EMT states. *In vitro* qRT-PCR was incorporated as an orthogonal expression-validation step for selected candidates. qRT-PCR further showed concordant upregulation of ULBP1, IL37, COL10A1, COL11A1, and IGF2BP3 in ATCC-derived bladder-cancer cell lines relative to SV-HUC-1, thereby providing orthogonal transcript-level support for the public-dataset observations.

## Data Availability

The publicly available transcriptomic datasets analyzed in this study are available from The Cancer Genome Atlas (TCGA-BLCA) and the Gene Expression Omnibus under accession numbers GSE13507 and GSE293189.
